# Proposing a Transactional Model of eHealth Literacy: Concept Analysis

**DOI:** 10.2196/10175

**Published:** 2018-10-02

**Authors:** Samantha R Paige, Michael Stellefson, Janice L Krieger, Charkarra Anderson-Lewis, JeeWon Cheong, Christine Stopka

**Affiliations:** 1 STEM Translational Communication Center College of Journalism and Communications University of Florida Gainesville, FL United States; 2 Department of Health Education & Behavior University of Florida Gainesville, FL United States; 3 Department of Health Education and Promotion East Carolina University Greenville, NC United States; 4 Department of Public Health University of Southern Mississippi Hattiesburg, MS United States

**Keywords:** eHealth literacy, Transactional Model of Communication, interpersonal communication, social media, mobile phone

## Abstract

**Background:**

Electronic health (eHealth) literacy was conceptualized in 2006 as the ability of internet users to locate, evaluate, and act upon web-based health information. Now, advances in eHealth technology have cultivated transactional opportunities for patients to access, share, and monitor health information. However, empirical evidence shows that existing models and measures of eHealth literacy have limited theoretical underpinnings that reflect the transactional capabilities of eHealth. This paper describes a conceptual model based on the Transactional Model of Communication (TMC), in which eHealth literacy is described as an intrapersonal skillset hypothesized as being dynamic; reciprocal; and shaped by social, relational, and cultural contexts.

**Objective:**

The objective of our study was to systematically examine eHealth literacy definitions, models, and measures to propose a refined conceptual and operational definition based on the TMC.

**Methods:**

Walker and Avant’s concept analysis method was used to guide the systematic review of eHealth literacy definitions (n=10), rating scales (n=6), models (n=4), and peer-reviewed model applications (n=16). Subsequent cluster analyses showed salient themes across definitions. Dimensions, antecedents, and consequences reflected in models and measures were extracted and deductively analyzed based on codes consistent with the TMC.

**Results:**

Systematic review evidence revealed incongruity between operational eHealth literacy included in definitions compared with literacies included within models and measures. Theoretical underpinnings of eHealth literacy also remain dismal. Despite the transactional capabilities of eHealth, the role of “communication” in eHealth literacy remains underdeveloped and does not account for physical and cognitive processing abilities necessary for multiway transactions.

**Conclusions:**

The Transactional Model of eHealth Literacy and a corresponding definition are proposed. In this novel model, eHealth literacy comprises a hierarchical intrapersonal skillset that mediates the reciprocal effect of contextual factors (ie, user oriented and task oriented) on patient engagement in health care. More specifically, the intrapersonal skillset counteracts the negative effect of “noise” (or impediments) produced by social and relational contexts. Cutting across health and technology literacies, the intrapersonal skillset of eHealth literacy is operationalized through four literacies that correspond with discrete operative skills: (1) functional (ie, locate and understand); (2) communicative (ie, exchange); (3) critical (ie, evaluate); and (4) translational (ie, apply).

## Introduction

Electronic health (eHealth) is increasingly being ingrained within the health care system and patient engagement experience. eHealth facilitates productive collaborations among informed patients, proactive health care professionals, and responsive health care systems to coordinate care for positive health outcomes [1,2]. Alongside the evolution of eHealth, patients can now interact with health information available on the internet and either synchronously or asynchronously exchange ideas, thoughts, and health-related data and media with other users through multimedia on computer-mediated platforms (eg, health information portals, personal health records, telemedicine apps, Web-based support groups or forums) [1,2]. Shaw et al [2] identified 3 overlapping domains of eHealth, including users’ interaction with technology, interaction with other users through mediated platforms, and use of information gained from these interactions to advance their health and well-being. As such, a core aspect of eHealth includes not only the use of technology but also the computer-mediated transaction of information among its users.

The Transactional Model of Communication (TMC) [3] posits that communication between two or more entities is dynamic, process oriented, and adapted or appropriated according to the context of the transaction. This context is shaped by the channel of communication (eg, telephone, email, letter), the source of communicators (eg, interpersonal, impersonal), language (eg, native, second), and the type of message (eg, mode of transmission, whether image, video, text, or other). Social, relational, and cultural contexts also drive the transactional process of communication. In the TMC, entities are not assigned roles as message “senders” or “receivers”; rather, their roles are interdependent, meaning that they are simultaneously message senders and receivers or simply communicators. Any person within a social situation is a communicator, whether his or her interaction is synchronous or asynchronous, verbal or nonverbal, and intentional or unintentional. In this model, communication extends beyond a simplistic view of message creation; the model views processing information as a vehicle for community and personal identity construction and impression management within the transactional context (eg, source, channel, message, language) [3-5]. The TMC functions under the assumption that interpersonal communication exists within a fluid state and that the transaction among communicators is constantly changing and mutually influenced.

The TMC can be extended to interpersonal computer-mediated communication (I-CMC). I-CMC occurs remotely with technology (eg, desktop computer, smartphone, tablet, laptop) through diverse message channels (eg, text, video, image) and sources (eg, personal friends and family, impersonal provider, peer) [6]. In the social era of eHealth, or Web 2.0, where two-way transactions occur among users, the device and channel drive the type and amount of information transmitted by diverse sources [7-9]. I-CMC notoriously fosters ambiguous communication because traditional in-person social and contextual cues that assist people in understanding the pragmatic meaning of messages are less salient across computer-mediated platforms [5,7]. With such cues filtered out, I-CMC can disrupt the accurate and smooth transmission of multimedia messages among communicators using various channels [5]. Similar to in-person communication, there are factors beyond contextual and social cues in the TMC that can exacerbate the ambiguity of message transmission via I-CMC.

Noise-inducing factors interfere with information transmission and accessibility among communicators, ultimately hindering their ability to access, understand, and transmit meaning to one another [10]. Noise-induced factors can be categorized as physical (ie, external factors), psychological (ie, mental and emotional belief-systems), physiological (ie, physical conditions, including auditory and verbal limitations, and medication effects), and semantic (ie, systems of meaning that do not correspond) [6]. With regard to I-CMC in the context of eHealth, these noise-inducing factors include technological usability challenges, stress or worry related to a recent disease diagnosis, exposure to scientific medical jargon, and physical limitations due to a health condition, just to name a few. Generally, “noise” can be compared with barriers or impediments widely published in the literature to describe hindrances to successful eHealth adoption and use [11-13]. In the TMC from the perspective of health-related I-CMC, however, barriers are operationalized beyond functional technological impediments; rather, they act as personal, relational, social, and cultural factors that hinder the process of communication [3,6]. As such, the high volume and constant flow of health information created and shared on the internet, coupled with the regular presence of noise-inducing factors, has the potential to attenuate users’ capacity to effectively and appropriately engage in the transmission of health-related communication. An essential aspect of successful transactional communication within computer-mediated contexts is the capacity to counteract the negative effects of noise.

To understand patients’ capacity to successfully use and benefit from eHealth, the concept of eHealth literacy was initially coined in 2006. eHealth literacy was defined as the ability to locate, evaluate, understand, and act upon health information from electronic sources [14,15]. Despite widespread use of this definition over the past decade, researchers have argued that this seminal construct and its corresponding eHealth Literacy Scale (eHEALS) are outdated because neither considers the evolving dynamic and social nature of eHealth [2,16-18]. In an attempt to synthesize eHealth literacy research and recommendations for its conceptual advancement, Griebel et al [19] posited that new eHealth literacy concepts do not build upon the assumptions and structure of existing models; rather, these models function in isolation and do not emanate from the existing literature. Moreover, empirical evidence shows that eHealth literacy definitions and models have insufficient theoretical underpinnings, which inhibits eHealth literacy researchers from developing an updated definition, model, and corresponding measure that reflects the social context of eHealth [19]. Together, these limitations perpetuate challenges in advancing our understanding of eHealth literacy in the social era of eHealth, specifically regarding its valid operationalization and measurement.

The purpose of this study is to propose a theoretical blueprint for defining and conceptualizing eHealth literacy in the transactional era of eHealth. Per the fundamental assumptions of the TMC, this study operationalizes eHealth literacy as an intrapersonal skillset grounded in counteracting the effect of noise during transactional interactions across computer-mediated platforms. In the context of I-CMC, the TMC is appropriate to form the basis of our proposed model because we aim to describe the communicative element of eHealth literacy and understand how underlying eHealth operational skills function in the larger context of computer-mediated transactions. In this study, we applied a concept analysis method, which is a rigorous method in which empirical literature is systematically surveyed to refine the operationalization of a construct [20,21]. The findings of this empirical review generate an operational definition and eHealth literacy model based on the TMC.

## Methods

### Sample and Procedures: Concept Analysis

**Figure 1:**
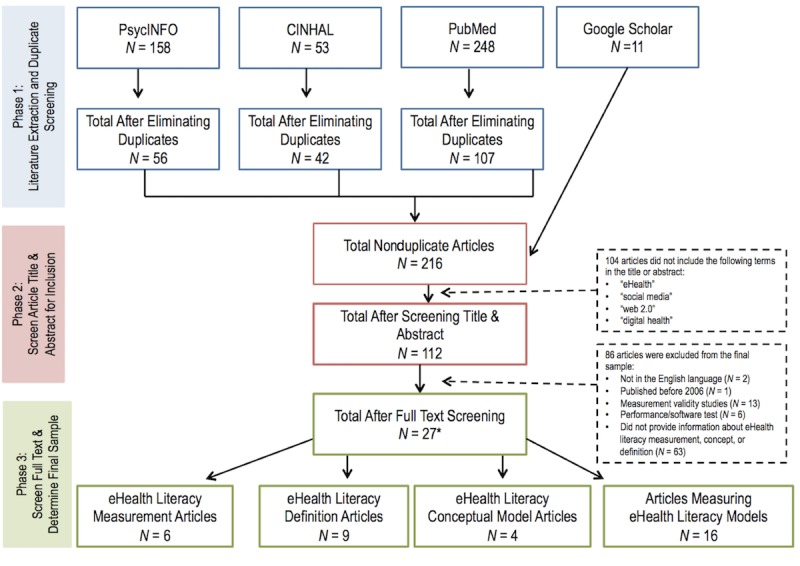
Literature extraction procedures. The asterisk indicates that Phase 3 N values exceed the total sample of 27 as some articles included both definitions and models or measures.

A series of keywords were combined with the Boolean operator (“AND”) and entered into 3 electronic databases (ie, PubMed, CINAHL, and PsycINFO). In each search query, a combination of 3 terms was entered to reflect the following: (1) *purpose* (ie, “concept,” “model,” “definition,” “framework,” “theory,” “measure,” “instrument,” “scale,” and “survey”); (2) *context* (ie, “eHealth,” “social media,” “Web 2.0,” “social network,” and “digital health”); and (3) *ability* (ie, “skill” and “literacy”). The same queries were conducted in Google Scholar to identify gray (or unpublished) literature. The final sample consisted of articles that (1) were published between 2006 and 2017 (2006 is the year that the seminal eHealth literacy definition, model, and measure were published); (2) were in the English language; (3) included the terms “eHealth,” “social,” “media,” “Web 2.0,” or “digital health” in the title or abstract; and (4) presented information on the concept, definition, or measurement of skills related to social media, digital health, and electronic health record use. Figure 1 presents literature review extraction procedures. Twenty-seven unique articles met eligibility criteria. Some articles included both definitions and models or measures; therefore, the asterisk indicates that Phase 3 N values exceed the total sample of 27.

Walker and Avant’s [21] concept analysis methodology was used to guide the data extraction and analysis procedures. Literature presenting definitions, antecedents, consequences, and attributes (ie, dimensions) of eHealth literacy was extracted. Articles that presented explicit definitions and conceptual models of eHealth literacy were considered. Moreover, the original sources of eHealth literacy empirical referents (ie, measurement instruments) were included in the final sample. Peer-reviewed empirical articles that included at least one of the models reviewed in the analyses were perused to identify information about antecedents and consequences of eHealth literacy.

### Data Analysis

An inductive analysis of eHealth literacy definitions was performed to identify thematic clusters from peer-reviewed and gray literature. Consistent with a separate concept analysis of health literacy [22], an inductive analysis of eHealth literacy definitions identified thematic clusters by *competence, contextual factors, action (operational behaviors or skills), object of interest,* and *objective*. The attributes of eHealth literacy conceptual models and measures were extracted and entered into a descriptive table where congruent components were identified; furthermore, antecedents (independent variables in analyses) and consequences (dependent variables) were extracted.

## Results

### Concept Analysis: eHealth Literacy Definitions, Models, and Measures

#### Existing Definitions of eHealth Literacy

Table 1 presents 10 eHealth literacy definitions published between 2006 and 2017. Seminal definitions solely focused on intrapersonal skills to access and use health information obtained from electronic sources [14,23]. Interactions between individual and technological factors became more salient in later definitions of eHealth literacy [17,24]. Chan and Kaufman [25], for example, posited that eHealth literacy is not solely dependent on cognitive processing; rather, it is influenced through interactions between cognition and technology. More recent definitions of eHealth literacy stated that eHealth skills function within the context of social, individual, and technological factors [16,19,26]. The interaction between diverse contextual factors and technological constraints influences eHealth skills and the ultimate capacity to improve health and wellness. Although implied in all definitions, one definition explicitly stated that eHealth literacy comprises a “hybrid of two other concepts,” including health literacy and technology literacies [27].

Textbox 1 presents the definitions of eHealth literacy into clustered themes. *Competence* is characterized as a set of skills and knowledge, predominantly referred to as “the ability.” *Influential Factors* that determine the said ability are characterized as the interplay between contextual factors (ie, individual and social) coupled with situational factors (ie, the type of health problem and type of technology). 

**Table 1:** Ten definitions of eHealth literacy from the research literature (2006-2017).

Article	Author	Year	Definition
1	Norman & Skinner [14]	2006	*The ability to seek out, find, understand and appraise, integrate, and apply what is gained in electronic environments toward solving a health problem.* [pg 2]
2	Bodie & Dutta [23]	2008	…*not just the ability to use the internet to find answers to health-related questions; it also entails the ability to understand the information found, evaluate the veracity of the information, discern the quality of different websites, and use the quality information to make informed decisions about health.* [pg 193]
3	Chan & Kaufman [25]	2011	*A set of skills and knowledge that are essential for productive interactions with technology-based health tools, such as proficiency in information retrieval strategies, and communicating health concepts effectively.* [pg 2]
4	Norman [17]	2011	*A foundational skill set that underpins the use of information and communication technologies for health.* [pg 1]
5	Neter & Brainin [24]	2012	*The ability of people to use emerging information and communication technologies to improve or enable health and health care.* [pg 1]
6	Paek & Hove [27]	2012	…*a hybrid of two other concepts, eHealth and health literacy, [in which] skills must be appropriate for the informational text people need to understand in their efforts to treat various health concerns.* [pg 728]
7	Werts & Hutton-Rogers [29]	2013	*The ability to gather and appropriately process health information retrieved online.* [pg 115]
8	Gilstad [26]	2014	*The ability to identify and define a health problem, to communicate, seek, understand, appraise, and apply eHealth information and welfare technologies*^a^*in the cultural, social and situational frame and to use the knowledge critically in order to solve the health problem.* [pg 69]
9	Bautista [16]	2015	*The interplay of individual and social factors in the use of digital technologies to search, acquire, comprehend, appraise, communicate, and apply health information in all contexts of health care with the goal of maintaining or improve the quality of life throughout the lifespan*. [pg 43]
10	Griebel et al [19]	2017	…*a dynamic and context-specific set of individual and social factors, as well as consideration of technological constraints in the use of digital technologies to search, acquire, comprehend, appraise, communicate, apply, and create health information in all contexts of health care with the goal of maintaining or improving the quality of life throughout the lifespan.* [pg 10]

^a^Welfare technologies: “strengthen a users’ independence, safety, control of surroundings, independent living and social activities, independent of age and disabilities” (pg 344) [28].

**Five clusters of eHealth literacy definitions from the literature. Numerals in brackets correspond with article numbers in Table 1.**

**Competence**

- Ability (1,2,5,7,8)
- Skills (6)
- Set of skills & knowledge (3)
- Foundational skillset (4)

**Contextual factors**

- Cultural, social, and situational frame (8)
- Interplay between social and individual factors in using technology (9)
- Dynamic and context-specific individual and social factors and technological constraints (10)
- To use information and communication technologies (4,5)

**Action**

- To locate
  - Find (1,2)
  - Seek (1,8)
  - Search (9,10)
  - Retrieve (3)
  - Gather (7)
  - Acquire (9,10)
- To understand
  - Comprehend (9,10)
  - Understand (1,2,6,8)
  - Process (7)
- To evaluate
  - Appraise (1,8,9,10)
  - Evaluate the veracity (2)
  - Discern the quality (2)
- To communicate (3,8,9,10)
- To create (10)
- To translate
  - Integrate (1)
  - Apply (1,8,9,10)
  - Use knowledge (8)

**Object of interest**

- Knowledge (1,8)
- Information
  - General (2,4)
  - Health (9,10)
  - Quality (2)
  - Emerging (5)
  - eHealth (8)
  - Online (7)
  - Text (6)

**Objective**

- For health (4)
- To address or solve a health problem (1,8)
- To make informed decisions about health (2)
- To improve or enable health and health care (5)
- To treat various health concerns (6)
- To maintain or improve the quality of life throughout the lifespan (9,10)

*Actions* are thematically clustered according to operational skills, including the capacity to locate, understand, evaluate, exchange, and apply or translate health information. The *Object of Interest,* or the purpose of performing the actions, includes obtaining knowledge from high-quality Web-based health information. Finally, the *Objective* of obtaining the object of interest is generally for the purposes of health enhancement or to maintain or improve the health-related quality of life throughout the lifespan.

#### Dimensions of eHealth Literacy

Since 2006, 4 models and 6 measurement instruments of eHealth literacy have been published; their purposes, guiding theoretical frameworks (if applicable), and dimensions used in describing the concept and measurement have been presented in Multimedia Appendix 1.

##### Conceptual Models (N=4)

Norman and Skinner’s [14] Lily Model posits that eHealth literacy is comprised of analytic skills central to the Web-based health information seeking experience, as well as context-specific skills that vary according to situation. In the Lily Model, health science and computer literacies are denoted as context-specific skills, whereas information, traditional literacy and numeracy, and media literacies are analytic-specific. As such, a high degree of eHealth literacy exists where individuals use context- and analytic-specific skills in concert, and it allows an individual to successfully achieve an eHealth goal.

Gilstad [26] adapted Norman and Skinner’s [14] Lily Model to describe how eHealth literacy mediates the effect of diverse contextual factors (or literacies) on productive patient-provider communication. This model posits that contextual “literacies” (ie, propositional, cultural, social, propositional, and procedural) coupled with situational factors (ie, type of health question and type of eHealth technology) directly impact context- and analytic-specific aspects of eHealth literacy as posed in Norman and Skinner’s Lily Model. In Gilstad’s model, the outcome associated with eHealth literacy is communicative expertise (ie, the capacity to discuss a personal or family concern with an offline health care provider).

Unlike Norman and Skinner [14] and Gilstad [26], Bautista [16] developed a model to posit that eHealth literacy is a process-oriented concept. In this model, Bautista states that eHealth literacy is reciprocal, meaning that this construct affects and is affected by diverse contextual and ecological factors. As such, Bautista defines eHealth literacy as comprising intrapersonal actions (ie, search, acquire, comprehend, appraise, communicate, and apply), the type of digital technology selected (ie, PC and mobile devices), the Web-based environment in which the search occurs (ie, social media vs traditional website), as well as the goal of using eHealth technologies (ie, maintenance and treatment) in particular health care contexts (ie, promotion, prevention, curative, and rehabilitation) across the lifespan.

Kayser et al [30] applied an informatics approach to conceptualize eHealth literacy through a multidisciplinary lens. This model applies a user-task-context matrix, grounded in health and digital literacy. The matrix is comprised of 7 elements from 3 domains (ie, user, task, and user-task). The model functions under the assumption that eHealth literacy is the degree of harmony between health care consumers’ needs and skills, as well as the capacity of the technology to meet those needs and foster those skills within the greater health care context.

Conceptual models have attempted to extend Norman and Skinner’s [14] Lily Model to depict how contextual factors influence individual eHealth skills. Gilstad [26] identified a number of situational, technological, and cultural factors that can influence the intrapersonal literacies outlined in the Lily Model. Kayser et al [30], who did not consider the Lily Model in their conceptualization of eHealth literacy, roughly defined influential contextual factors as user- and task-domains, positing that eHealth skills are dependent on both the situation and person. Only Bautista’s [16] model depicts operational skills that include “communication” as a central skill. Bautista’s model also depicts eHealth literacy as intrapersonal skills that have reciprocal relationships with contextual factors, the type of technology, personal factors (ie, age), and the purpose of the eHealth experience.

Synthesized together, these 4 models suggest that eHealth literacy is an intrapersonal skillset shaped by diverse contextual factors influencing the user and the situation prompting the eHealth interaction.

##### Measurement Instruments (N=6)

Multimedia Appendix 1 also presents the results of 6 self-administered eHealth literacy rating scales, including their purposes, guiding frameworks, and dimensions. Norman and Skinner’s [15] eHEALS was the seminal eHealth literacy measurement instrument, developed as a unidimensional scale grounded in self-efficacy to reflect the Lily Model [14]. Chew and Yuqian [31] refute that the eHEALS reflects dimensions of the Lily Model. Their measure identified items from the Health Information National Trends Survey to address each of the literacies within the Lily Model. Unfortunately, insufficient evidence was reported to support the psychometric properties of their generic eHealth literacy measure.

Other measurement instruments assessed motivation or readiness to use eHealth. Through formative focus groups with older adults with chronic diseases, Koopman et al [32] derived 8 dimensions of eHealth motivation, including (but not limited to) the need for health information, preferred mode and channel of eHealth interaction, and electronic privacy, but these dimensions were not based on an existing conceptualization of eHealth literacy. Bhalla et al [33] assessed eHealth readiness by conducting formative research with eHealth consumers to identify themes that corresponded with the constructs of Social Cognitive Theory, specifically self-regulation to use eHealth. Despite being grounded in a health behavior theory, the dimensions included within this measure do not reflect the central components of a proposed or existing eHealth literacy model.

Most recently, measurement instruments have been developed to account for the social features of eHealth. Seçkin et al [34] identified 3 important concepts from a systematic literature review of health literacy that appeared to be central to eHealth literacy, including cognitive literacy (trust), interactional literacy (communication with offline health care providers), and behavioral literacy (apply learned health behaviors). Additionally, van der Vaart and Drossaert [35] developed an instrument to measure digital health literacy among rheumatic patients. The items on this scale capture dimensions about the capacity to use technology, navigate Web-based health information, create text messages for other users, and take precautions to protect the privacy of themselves and other users. These most recent measurement instruments begin to consider the operational skills related to eHealth proficiency; however, these measures are derived from formative research with limited application to eHealth literacy definitions or conceptual models.

#### Antecedents and Consequences of eHealth Literacy

Functional (or basic), health, and technology literacies are fundamental to eHealth skills [14,24,29,36,37]. Antecedents that influence eHealth skills include personal, relational, knowledge, and technological determinants. *Personal Determinants* influencing eHealth literacy include income and education [15,18,24,36-39], race or ethnicity [39], gender [40], age [18,24,40-42], marital status [40], and health status [41]. *Relational Determinants* include social influences or norms and alleviated linguistic and cultural barriers to health information [27,29]. *Knowledge Determinants* include the type and amount of health information preferred and the amount of pre-existing knowledge about the health concern [14,24,30,40]. *Technological Determinants* include motivation to use technology for health [15,23,24,27,36,42], access to technological devices [26,43], the type and number of technologies used to access health information [15,18], frequency of using eHealth [24,27,41,44], and preference for using eHealth to help address a particular concern [37].

The consequences of eHealth literacy primarily comprise intrapersonal factors, which have a residual effect across socioecological contexts. The most salient intrapersonal consequence includes a change in the degree of patient engagement [14,29,45]. People with a high degree of eHealth literacy report greater health care access [42]; better health-related outcomes [29,36,43]; and participation in proactive health behaviors offline, including self-management behaviors [24], patient-provider communication [24], and cancer screenings [40]. Consistent with the central tenants of eHealth literacy, a greater degree of confidence in eHealth skills was associated with higher self-reported comprehension [46], critical evaluation [44,45], and trust in Web-based health information from diverse sources and channels [26,39]. Positive self-reported eHealth skills predict motivation to continue using eHealth [23,26], particularly because it is perceived as a useful tool for supplementing health care [44].

### Transactional Model of eHealth Literacy

This synthesis and review of eHealth literacy definitions, models, and measures posits that the multidimensional construct is a reciprocal intrapersonal skillset influenced by the interaction between user- and task-oriented factors, which drive patient engagement, empowerment, and informed decision making. This is consistent with the theoretical underpinnings of the TMC [3]. According to the assumptions of the TMC [3], information transaction is dependent on the interaction between a series of contextual factors. In synthesizing the antecedents of transactions based upon eHealth literacy literature and the TMC, the contextual factors that influence eHealth literacy can be categorized as task-oriented features (ie, message type, source, channel, and language) and user-oriented features (ie, personal, relational, knowledgeable, and technological). The interactions between task- and user-oriented factors produce variable degrees of physical, semantic, psychological, and physiological noise [3,6,10]. The effects of the noise can either hinder or facilitate successful transaction of Web-based health information. The intrapersonal skillset of eHealth literacy will be integral for a user to benefit from the eHealth experience. Per eHealth literature, it is probable to hypothesize that the eHealth experience will inform the perceived affordance of eHealth in the future. This reciprocal feature of our model further captures the transactional and continuous elements of eHealth.

Existing definitions and models do not capture the transactional nature of eHealth literacy, specifically regarding information exchange, knowledge application, and message generation when communicating with other users. The most recent eHealth literacy frameworks depict eHealth literacy according to the intersection of user attributes, perceived motivation or control, and experiences using eHealth [30,47]. This framework posits that the ability of an individual to actively engage with digital services is central to the eHealth experience. The operationalization of these abilities, however, appears limited to functional skills related to eHealth or to comfort in handling technologies to learn about health information or enter health-related data [30,46,47]. To build upon these previous frameworks, our proposed definition and model of eHealth literacy considers technology a tool that is used as a vehicle to help users access and exchange health information that can be critically analyzed and translated across socioecological facets of health care. We extend beyond these basic functional behaviors related to technology readiness and engagement and rather consider eHealth literacy as a hierarchical skillset that allows people to not only use technology but also engage with others via technology to participate in dynamic health information seeking and transactional exchanges across computer-mediated platforms. The contribution of our proposed definition and model lies in its ability to position existing eHealth literacy literature through a translational health communication lens. We aim to theoretically capture the transactional nature of eHealth, specifically regarding both technology use and social engagement. The theorized definition and model are proposed as follows.

#### Proposed Definition

The following definition of *eHealth literacy* is proposed:

The ability to locate, understand, exchange, and evaluate health information from online environments in the presence of dynamic contextual factors and to apply the knowledge gained across ecological levels for the purposes of maintaining or improving health.

This definition builds upon previous eHealth literacy definitions and extends the concept to the context of the TMC. First, operational skills comprise and correspond with the central aspects of eHealth [2]: (1) interaction with technology (ie, *locate, understand*); (2) interaction with other users through mediated platforms (ie, *exchange*); and (3) assessment (ie, *evaluate*) and action (ie, *apply*) for health advancement. Second, the proposed definition acknowledges that eHealth literacy is highly contextual as it varies according to the interplay between user- and task-oriented factors. Third, this definition highlights the affordances of technology, which assist lay end users to access and exchange health information using electronic tools. Based on the synthesis of the literature, proficiency in using a technology or a Web-based environment does not solely determine one’s eHealth literacy; rather, it is the capacity of the users to achieve their intended eHealth goals when encountering noise that challenges the successful use of technology and transaction of health information. Finally, consistent with interpersonal communication literature and the TMC [3,5,6], this definition clarifies that “communication” in eHealth literacy is the ability to construct relationships and identities with other Web-based users through health information exchange.

Through this updated definition of eHealth literacy, we extend beyond functional behaviors related to technology readiness and engagement. We consider eHealth literacy as a hierarchical skillset that not only allows users to engage with technology but also engages other users via technology for dynamic health information seeking and transactional exchanges across computer-mediated platforms. The contribution of our proposed definition lies in its ability to position current eHealth literacy literature through a translational health communication lens.

#### Proposed Model

**Figure 2:**
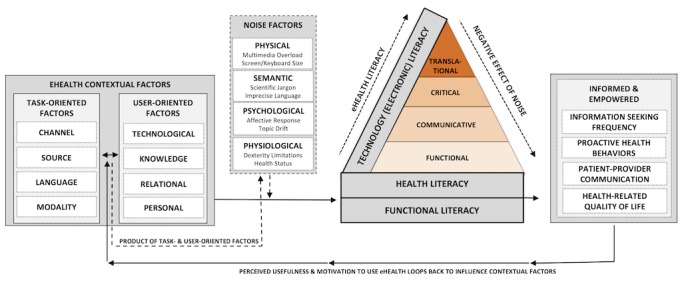
The Transactional Model of eHealth Literacy.

Consistent with the definition above, Figure 2 presents the Transactional Model of eHealth Literacy (TMeHL), which is derived from a systematic review of the literature (ie, concept analysis) and is theoretically based on the TMC. This model does not specify encoders (sender) and decoders (receiver); rather, it treats the communication transaction as a continuous process that is constantly modified according to diverse eHealth contextual factors and prior eHealth experiences. The TMeHL consists of 3 assumptions: (1) task-oriented and user-oriented factors interact to produce physical, semantic, psychological, and physiological noise during the transaction process; (2) eHealth literacy, a multidimensional and hierarchical intrapersonal skillset, counteracts the effect of noise on the transaction; and (3) patient engagement is reciprocal and influences future interactions between eHealth contextual factors and their effect on eHealth literacy. Although the primary consequence associated with eHealth literacy is being an informed and engaged patient across diverse socioecological contexts, there is no “end goal” of eHealth literacy. The capacity of an informed and engaged patient to apply knowledge gained from an eHealth transaction across diverse socioecological factors (ie, trust in eHealth, productive patient-provider communication, greater eHealth use and perceptions of its usefulness, and positive health-related quality of life) will ultimately inform patients’ future eHealth motivation and perceived usefulness for addressing a particular health concern. In turn, these experiences are hypothesized to inform future experiences and perceived affordances of eHealth by shaping task- and user-oriented factors that drive future noise production and eHealth skills.

Consistent with prior eHealth literacy models [26,30], a series of task-oriented and user-oriented factors comprise the eHealth context. However, these factors do not function in isolation, and they extend beyond the ability to interact and use technology; rather, these factors interact with one another to shape the transactional process of eHealth experience, including eHealth intrapersonal skills. *Task-Oriented Factors* include the channel in which the transaction occurs (eg, social media, electronic health record, email), the source or identity of the communicators (eg, peer, friend, family member, health care provider), the language used to communicate (eg, native, second), and the modality of the message (eg, image, text, video). *User-Oriented Factors,* however*,* comprise factors that are central to the user, rather than to the situation or task. These factors include personal demographic information, including education, gender, and age. Relational support is described as the amount of support or perceived social norm in using eHealth. 

The degree of pre-existing knowledge about the health topic and the desire to obtain more information is also a user-oriented factor. Finally, there is a technological user-oriented factor, which is a general assessment of the users’ access, preference, and frequency of use. Consistent with the TMC [3], the interactions between task- and user-oriented factors produce external stimuli, or “noise,” which can serve as a hindrance or facilitator to the transaction.

Noise-inducing factors in the TMC, as well as other interactional communication models, comprise physical, psychological, physiological, and semantic factors [3,6,10]. Although evidence shows that diverse internal and external factors hinder and facilitate the capacity of eHealth users to successfully achieve their intended goals on the internet [48-50], no other eHealth literacy model or measurement instrument reviewed in this study considered the concept of “noise” as being part of the eHealth experience, beyond contextual eHealth factors. *Physical Noise* can include external factors that hinder the eHealth experience, including cognitive or information overload due to a wide variety of multimedia or physical challenges with the technology used (eg, screen size is too small or not bright enough, keyboards are too small). *Psychological Noise* includes the affective response to the eHealth experience, including the urgency for the information or the nature of the search (eg, cancer clinical trials vs physical activity information). *Physiological Noise* can be either temporal or permanent, meaning that it could be dexterity limitations due to a health condition or pain from a briefly debilitating migraine. Finally, *Semantic Noise* is the disagreement between meaning systems, including excessive use of scientific or wordy jargon from one or more communicators, as well as use of emojis or emoticons to transmit information. Ultimately, the degree of noise in a computer-mediated transaction is produced by the interaction between these task- and user-oriented factors.

The intrapersonal eHealth literacy (shown in Figure 2 as the hierarchical triangle) mediates the relationship between the effect of noise on eHealth contextual factors and the degree to which an eHealth end user is informed and empowered. Theoretically, eHealth literacy has an inverse relationship with the negative effect of noise. In other words, greater eHealth literacy negates the detrimental effects of noise produced from eHealth contextual factors and promotes a positive eHealth experience. This is consistent with the evidence that greater frequency of using eHealth improves proficiency in Web-based health information seeking [24] as users become more familiar with their information needs, the technology, and the usefulness of eHealth.

The intrapersonal skillset of eHealth literacy may be grounded in 3 foundational elements: Functional Literacy, Health Literacy [51], and Technology Literacy [52]. *Functional Literacy*, or the basic reading and writing skills [53], is a basic predecessor of both health and technology literacy. Together, health and technology literacy are central to eHealth skills [23]. The most recent definition of *Health Literacy* posits that it is [22]…

…linked to literacy and entails people’s knowledge, motivation, and competence to access, understand, appraise, and apply health information in order to make judgments and take decisions in everyday life concerning health care, disease prevention, and health promotion to maintain or improve the quality of life during the life course.

pg 3

Technology Literacy is more concretely defined as “the ability to use, manage, assess, and understand technology” (pg 9) [52]. Without proficiency in functional literacy, eHealth users would not be able to successfully function within a health care context, let alone use technological devices or computer-mediated environments to address a health inquiry.

In our model, health and technology literacies shape a multidimensional and hierarchical intrapersonal skillset, which comprises 4 eHealth literacies. These eHealth literacies are aligned with the gold-standard health literacy model [53,54] to include *Functional*, *Communicative*, *Critical*, and *Translational eHealth Literacies*. Existing eHealth literacy definitions, models, and measurement instruments include a high volume of literacies and minimal insight into their relationship with underlying skillsets. Empirical evidence has hinted that the scientific community should consider reeling eHealth literacy conceptualizations back to seminal 4-tiered operational behaviors or literacies prevalent in health literacy and general literacy literature. This has been shown in a recent library of research on eHEALS, the seminal model of eHealth literacy, positing that it is not a unidimensional measure; rather, it may be a measure of 2-3 constructs that assess eHealth awareness, Web-based health information seeking skills, and evaluation or application skills [55-57]. The TMeHL builds on these principles to define 4 eHealth literacies.

The eHealth literacies presented in Figure 2 capture the hierarchical nature of these unique yet related skills, which map to operational skills proposed in our refined definition of eHealth literacy. Consistent with the health literacy literature [53,54], functional eHealth literacy is a foundational skill that precedes the remaining literacies. This literacy comprises lower-level operational skills, including the ability to locate and understand health information. Translational eHealth literacy is located at the highest level, as being proficient in this top-tier literacy requires a degree of proficiency to be present across all lower-level literacies. This hierarchical depiction shows that lower-level literacies and operational skills represent the necessary building blocks to achieve optimal proficiency in the higher-level literacies and operational eHealth skills. Stated differently, an eHealth lay end user must have basic skills in reading and writing and in typing to successfully exchange, evaluate, and apply health information from the internet. Each of these literacies is described and operationalized below, alongside the corresponding behaviors outlined in the proposed definition.

##### Functional eHealth Literacy (Operational Behaviors: to Locate and Understand)

According to Nutbeam [53], the definition of functional health literacy, which was adapted by Freebody and Luke [58], describes having:

Sufficient basic skills in reading and writing to be able to function effectively in everyday situations.

pg 263

Considering the technological context of functional health literacy, it is important to determine how well an individual can successfully read and write about health via a technological device. Therefore, *Functional eHealth Literacy* is defined as:

Basic skills in reading and writing (typing) about health to effectively function on the internet.

##### Communicative eHealth Literacy (Operational Behavior: to Exchange)

Communicative literacy is [53]:

Advanced cognitive and literacy skills, which together with social skills, can be used to participate in everyday activities to extract information and derive meaning from different forms of communication and to apply new information to changing circumstances.

pg 264

In its original conceptualization, communicative literacy was intended to assess patients’ communication skills when engaging with offline health care professionals [53,54]. eHealth is a computer-mediated form of communication, which provides limited salience to social and nonverbal cues [6]. According to Spitzberg and Cupach [59], success in achieving instrumental, self-presentation, and/or relational goals is determined based on the degree that interpersonal communication is appropriate and effective. Appropriate communication is consistent with social norms and relationships (stranger or close friend) among communicators. Effective communication helps achieve the desired goal of the health information seeking experience and interaction. There are 3 fundamental interpersonal communication skills that guide the degree to which someone is communicating appropriately and effectively [59]: (1) *control*, effectiveness in managing a situation to negotiate interpersonal problems and achieve a communicative goal; (2) *collaboration*, adhering to social norms to achieve an interaction goal; and (3) *adaptability*, acclimating to challenges by improvising communicative styles based on contextual and social cues. These principles of interpersonal communication are also consistent with the underlying elements of participatory media that foster collaboration, openness, participation, and apomediation (Web 2.0), which differs from more static and linear, one-way information seeking behaviors (Web 1.0) [60]. As such, integrating interpersonal communication competence into eHealth literacy represents a unique contribution to understand the social aspects of eHealth. In our proposed model, *Communicative eHealth Literacy* is defined as:

The ability to collaborate, adapt, and control communication about health with users on social online environments with multimedia.

##### Critical eHealth Literacy (Operational Behavior: to Evaluate)

Critical literacy is defined as [53]:

Advanced cognitive skills, which together with social skills, can be applied to critically analyze information and to use this information to exert greater control over life events and situations.

pg 264

Through the lens of the TMC, critical eHealth literacy includes being aware of the type of health information that is communicated to and from Web-based users, as well as the source from which this information is presented. This includes not only source and information credibility but also entails evaluating the relevance of and risks related to sharing personal information with Web-based sources through diverse channels. In this model, *Critical eHealth Literacy* is defined as:

The ability to evaluate the credibility, relevance, and risks of sharing and receiving health information on the internet.

##### Translational eHealth Literacy (Operational Behavior: to Apply)

Developing a concept that acknowledges the dichotomy between “what people know” and “what people do” represents a fundamental gap in the health literacy literature [61]. Translating knowledge gained through a health-related interaction is the “process of moving what we learned…to the actual application of knowledge in a variety of practice settings and circumstances” [62]. In public health research, knowledge translation is a systems-level approach to transforming knowledge gained from rigorous research on societies for improved health outcomes [62,63]. Within the context of eHealth, health information seekers often adopt the role of lay health researchers as they become exposed to new information including health-related knowledge from diverse sources (eg, peers, family, providers) and communication channels (eg, social media, electronic health records, news outlets). Strategies used to determine the applicability of new health information for translation into our existing knowledge structures depend on contextual factors, including personal and situational contexts [64]. This process likely depends on the skills a person has to identify and on the implementation of successful strategies for translating health information gained from electronic sources. Based on existing literature on knowledge translation, we propose the dimension “translational eHealth literacy.” *Translational eHealth Literacy* is defined as:

The ability to apply health knowledge gained from the internet across diverse ecological contexts. Translational literacy is the highest cognitive level of eHealth literacy, meaning it is informed and built upon from all lower-level eHealth literacy dimensions (ie, critical, communicative, and functional).

## Discussion

### Principal Findings

In this study, we systematically reviewed literature on eHealth literacy to provide an updated understanding of what we know about the construct in today’s more transactional era of eHealth. Unlike Griebel et al [19], who suggested that eHealth literacy literature functions in solidarity and does not build upon prior literature, the results of this systematic review suggest that eHealth literacy literature has gradually built upon existing definitions and models to extend the construct to account for the evolving nature of eHealth. Unfortunately, while progress has been made, results from this concept analysis illustrate that existing literature’s attempts continue to miss capturing the transactional nature of eHealth, specifically the skills needed to thrive within Web-based environments where social and contextual cues to action are limited. Instead, literature over the past decade has explored basic technological and contextual factors that influence individual eHealth literacy, but this work has provided little insight into transactional implications of eHealth literacy and intrapersonal skills that are important for cultivating positive eHealth experiences in various contexts. The intrapersonal skillset of eHealth literacy remains underdeveloped, especially regarding the role of communication. Results from this study were used to present a theoretical proposition of eHealth literacy that supports the transactional elements of eHealth. Subsequently, this new knowledge is leveraged to generate a refined definition of eHealth literacy and its complementary model.

Existing eHealth literacy definitions include operational skills required for an eHealth end user to thrive on the internet (ie, locate, understand, evaluate, apply, and, most recently, communicate or create). However, dimensions of existing models and measures are not intuitively aligned with the intrapersonal operational skills outlined in their corresponding definitions. Norman and Skinner’s [15] eHEALS was intended to serve as a unidimensional scale to capture the Lily Model’s eHealth literacy, or the self-efficacy to locate, understand, evaluate, and act upon Web-based health resources [14]. Over the past decade, strong empirical evidence has shown that eHEALS is a three-dimensional measure that assesses eHealth users’ self-efficacy in their eHealth awareness, information seeking, and evaluation and actions related to Web-based health information [55-57]. This research begins to clarify the relationship between operational skills outlined in eHealth literacy definitions and dimensions captured in corresponding measurement instruments. Moreover, limited empirical attention has been paid to the transactional operational skills needed to thrive within the social era of eHealth. The proposed TMeHL seeks to bridge this fundamental disconnect in the eHealth literacy literature by proposing a definition and model that specify important operational skills and literacies that should be considered.

The dimension of “communication” was significantly underdeveloped in eHealth literacy definitions, models, and measurement instruments reviewed. “Communication” was not integrated within definitions until 2011, and it first appeared in a conceptual model in 2014 as an outcome related to high eHealth literacy, not as an integral or defining element [26]. Communication was not considered a core element of eHealth literacy; rather, existing measures stressed the importance of “interactivity,” or the ability to talk about findings from an internet search with an offline health care provider [32,34]. The most recent measurement instrument operationalizes “communication” as the ability to self-create, add, or generate messages on social media with a technological device [35]. Interestingly, in the most recent definition, Griebel et al [19] posited that “communicating” and “creating” are two discrete skills. The role of communication appears to be having an identity crisis in eHealth literacy literature. In the TMC, particularly in computer-mediated contexts, communication is a vehicle that facilitates the process of cocreating information within diverse contexts among two or more communicators [3,5]. The proposed TMeHL definition and model consider communication as a central skill of eHealth literacy that affects critical (evaluative) and translational (application) elements of the eHealth experience. Researchers should view communication in this manner, rather than as an end goal or single act of generating a health-related message.

### Limitations

This study is not without limitations. Despite the rigorous extraction procedures and inclusion of gray literature, it is possible that not all eHealth literacy models, definitions, and measures were included because of the time frame of our literature extraction procedures. A qualitative approach was used to extract and analyze the literature in this study, which is prone to researcher bias [65]. However, we applied a concept analysis method [20,21], which is a rigorous and well-regarded approach to refine and operationalize an evolving concept, like eHealth literacy. This study proposes a refined definition and model of eHealth literacy to assist researchers and practitioners in “keeping up” with the evolving nature and transactional approaches currently ingrained in eHealth. We present a theoretical proposition of eHealth literacy that supports the transactional elements of eHealth.

The theoretical tenants from TMC that were used to derive the TMeHL were informed by results of previously published systematic reviews, theory-driven articles, measurement development studies, and empirically driven original research articles examining eHealth literacy in diverse populations. Published research informed development of TMeHL, but the model has not been subjected to any formal evaluation or hypothesis testing. Per recommendations by Griebel et al [19], future research is needed to obtain key stakeholder feedback about eHealth literacy models. Formative validation based on stakeholder input will provide an additional layer of validity evidence to support model testing in quantitative studies. Specifically, there is a need to explore how eHealth literacy serves as a mediator to counteract the negative effects of noise in eHealth transactions, as described in this study.

### Conclusion

Existing eHealth literacy definitions, models, and measures do not account for the transactional nature of eHealth. Few have sufficient theoretical underpinnings. Prior to our contribution, researchers had yet to capture the “social” elements of eHealth with theoretical underpinnings from the perspective of transactional communication. This is primarily due to the high volume of overlapping and inconsistent literacies, as well as the underdeveloped nature of “communication” as an integral component of eHealth literacy. In addition, existing eHealth literacy definitions, models, and measures failed to adequately integrate “communication” as an essential component of eHealth literacy. Because of this, a refined eHealth literacy definition and model based on the TMC are proposed. There is a need to validate this model with key stakeholders in eHealth and test the assumptions of the model with eHealth experts and lay end users.

## Appendix

Multimedia Appendix 1 Dimensions (content areas) of eHealth literacy conceptual models and measures.

## Abbreviations

eHealth: electronic health
eHEALS: Electronic Health Literacy Scale
I-CMC: interpersonal computer-mediated communication
TMC: Transactional Model of Communication
TMeHL: Transactional Model of eHealth Literacy
