# Supplementary material for: Proposing a Transactional Model of eHealth Literacy: Concept Analysis
Source: J Med Internet Res. 2018 Oct 2;20(10):e10175. doi: 10.2196/10175 (PMC6231800; doi:10.2196/10175)
Supplement: Multimedia Appendix 1 [file jmir_v20i10e10175_app1.pdf]

Multimedia Appendix 1. Dimensions (content areas) of eHealth literacy conceptual models and measures.

**Background Information**	**Dimensions (Content Areas)**
**Conceptual Models**	
**Author:** Norman & Skinner [14]<br><br>**Name**: Lily Model<br><br>**Purpose**: To develop a model that accounts for the unique attributes of health literacy in an electronic/networked world.<br><br>**Guiding Framework:** Consumer eHealth	Context-Specific Literacies<br>• Health literacy *(ie, basic literacy in the context of the health*<br>• Science literacy (*ie, understand the scientific process)*<br>• Computer literacy *(ie, use computers to solve issues)*<br>Analytic-Specific Literacies<br>• Traditional literacy/numeracy (*ie, basic or prose literacy)*<br>• Information literacy *(ie, resource awareness)*<br>• Media literacy *(ie, critical thinking)*
**Author:** Gilstad [27]<br><br>**Name**: A comprehensive model of eHealth Literacy<br><br>**Purpose**: To develop a comprehensive model of eHealth literacy that accounts for the competencies and analytic notions of what makes a user "eHealth literate."<br><br>**Guiding Framework:** Extends from Norman & Skinner's Lily Model; Self-Efficacy Theory	Contextual Factors<br>• Propositional literacy *(ie, learning through facts, theory)*<br>• Cultural literacy *(ie, cultural knowledge)*<br>• Social literacy *(ie, norms, values, rules, and regulations)*<br>• Procedural literacy *(ie, experiential or physical learning with*<br>• Contextual literacy *(ie, understanding the social and health*<br>Situational Factors<br>• Type of health question<br>• Type of eHealth technology<br>Context-Specific Literacies<br>• Health literacy *(ie, basic literacy in the context of the health*<br>• Science literacy *(ie, knowledge of the scientific method)*<br>• Computer literacy *(ie, knowledge about using a computer a*<br>Analytic-Specific Literacies<br>• Traditional literacy/numeracy *(ie, understanding, communic*<br>• Information literacy *(ie, using information to identify and so*<br>• Media literacy *(ie, analyze visual and audio information)*<br>Communicative Expertise *(ie, interaction with an offline healthcare*
**Author:** Bautista [16]<br><br>**Name**: N/A[a]<br><br>**Purpose**: To develop a conceptual model of eHealth literacy that can guide operational measures.<br><br>**Framework**: Health & Digital Literacy	• Contextual factors *(ie, social and individual factors)*<br>• Lifespan *(ie, age group)*<br>• Quality of life *(ie, maintenance and improvement)*<br>• Healthcare context *(ie, health promotion, prevention, cure, reha*<br>• Actions *(ie, search, acquire, comprehend, appraise, communicat*<br>• Use of technologies *(ie, PC, mobile device, Internet, social medi*

**Author:** Kayser et al. [30]<br><br>**Name**: Expanded User-Task-Context Matrix for eHealth Literacy<br><br>**Purpose**: To conceptualize eHealth literacy of Health IT Systems.<br><br>**Framework**: User-Task-Context Matrix	• Health and digital literacy<br>• Healthcare context<br>• User Domain<br>• Knowledge about one's health<br>• Ability to interact with information<br>• Ability to engage with technology<br>• Task Domain<br>• Access to technologies that work<br>• Access to technologies that suit individual needs<br>• User-Task Domain<br>• Feel that using technology is beneficial to address a health [illegible]<br>• Feel in control and secure in using a technology to address [illegible]
<u>**Measurement Instruments**</u>	
**Author:** Norman & Skinner [15]<br><br>**Name**: eHealth Literacy Scale (eHEALS)<br><br>**Purpose**: To assess knowledge, comfort, and perceived skills at finding, evaluating, and applying electronic health information to health problems.<br><br>**Framework:** Reflects the Lily Model, but items do not correspond with model literacies.	• Locate electronic health information<br>• Evaluate electronic health information<br>• Apply knowledge gained from electronic health information
**Author:** Koopman et al [31]<br><br>**Name**: Patient Readiness to Engage in Health Internet Technology (PRE-HIT)<br><br>**Purpose**: To measure readiness or motivation, a key aspect of "competence," to use eHealth.<br><br>**Framework:** Dimensions were derived from focus groups with chronic disease patients	• Health information need<br>• Internet experience<br>• Computer anxiety<br>• Preferred mode of interaction<br>• Relationship with doctor<br>• Cell phone expertise<br>• Online privacy<br>• "No news is good news"
**Author:** Chew & Yuqian [32]<br><br>**Name**: eHealth Literacy<br><br>**Purpose:** To measure eHealth literacy as the ability to use information technology to search, locate, process, and understand health information to improve health and healthcare.<br><br>**Framework**: Included items from the Health Information National Trends Survey (HINTS) that	• Health literacy<br>• Science literacy<br>• Media literacy<br>• Computer literacy<br>• Traditional literacy/numeracy<br>• Information literacy

align with core literacies of the seminal Lily Model.	
**Author:** Bhalla et al [33]<br><br>**Name**: eHealth Readiness Scale<br><br>**Purpose:** To assess user preparedness to engage in eHealth interventions.<br><br>**Framework:** Bandura's Social Cognitive Theory (SCT)	• Self-motivation and self-regulation in eHealth task completion
**Author:** Seçkin et al [34]<br><br>**Name**: e-Health Literacy Scale (e-HLS)<br><br>**Purpose:** To develop an eHealth literacy scale that assesses how well people provided digital health content can discern high quality from low-quality information.<br><br>**Framework:** eHealth literacy is grounded in the concept of health literacy, thus dimensions were derived from a review of the literature	• Cognitive literacy *(ie, trust in the credibility of web-based health*<br>• Interactional literacy *(ie, communication with offline health care*<br>• Behavioral literacy *(ie, action related to evaluating and appraisi*<br>*information)*
**Author:** van der Vaart & Drossaert [35]<br><br>**Name**: Digital Health Literacy Instrument (DHLI)<br><br>**Purpose**: To develop and measure a self-reported and performance-based measure Web 1.0 and Web 2.0 skills.<br><br>**Framework:** Dimensions were derived from formative research examining the eHealth performance of patients with rheumatic diseases	• Operational skills *(ie, to use the computer and Internet)*<br>• Navigation skills *(ie, to navigate and orient on the Internet)*<br>• Information searching skills *(ie, to use correct search strategies)*<br>• Evaluation skills *(ie, to evaluate the reliability and relevance of v*<br>• Self-creation skills *(ie, to add self-generated content to web-base*<br>• Privacy protection skills *(ie, to protect and respect privacy while*

N/A: not applicable.
